# Non-Newtonian Droplet Generation in a Cross-Junction Microfluidic Channel

**DOI:** 10.3390/polym13121915

**Published:** 2021-06-09

**Authors:** Maryam Fatehifar, Alistair Revell, Masoud Jabbari

**Affiliations:** Department of Mechanical, Aerospace and Civil Engineering, The University of Manchester, Manchester M13 9PL, UK; maryam.fatehifar@postgrad.manchester.ac.uk (M.F.); alistair.revell@manchester.ac.uk (A.R.)

**Keywords:** microfluidics, droplet, cross-junction, non-Newtonian, power-law, CFD

## Abstract

A two-dimensional CFD model based on volume-of-fluid (VOF) is introduced to examine droplet generation in a cross-junction microfluidic using an open-source software, *OpenFOAM* together with an *interFoam* solver. Non-Newtonian power-law droplets in Newtonian liquid is numerically studied and its effect on droplet size and detachment time in three different regimes, i.e., squeezing, dripping and jetting, are investigated. To understand the droplet formation mechanism, the shear-thinning behaviour was enhanced by increasing the polymer concentrations in the dispersed phase. It is observed that by choosing a shear-dependent fluid, droplet size decreases compared to Newtonian fluids while detachment time increases due to higher apparent viscosity. Moreover, the rheological parameters—*n* and *K* in the power-law model—impose a considerable effect on the droplet size and detachment time, especially in the dripping and jetting regimes. Those parameters also have the potential to change the formation regime if the capillary number (Ca) is high enough. This work extends the understanding of non-Newtonian droplet formation in microfluidics to control the droplet characteristics in applications involving shear-thinning polymeric solutions.

## 1. Introduction

**Figure 1:**
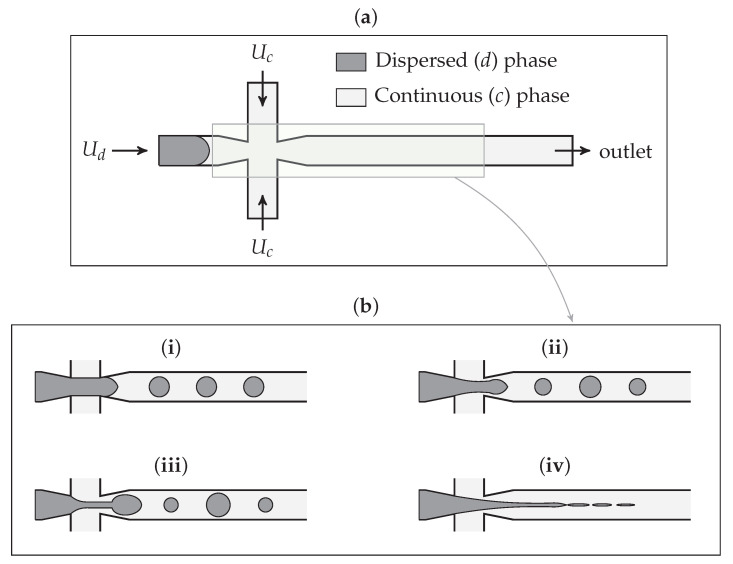
(**a**) Flow-focusing microfluidic channel; (**b**) schematic of different regimes in flow-focusing microfluidics: (**i**) squeezing, (**ii**) dripping, (**iii**) jetting and (**iv**) tip-streaming.

Microfluidic technology (c.f. Figure 1a), capable of controlling and manipulating minuscule amounts of fluid in micrometre channels, has attracted researchers since the start of the 21st century [1], and then gained broad attention after 2010 due to its promising potential to be used in biomedical and biochemical studies. Among many applications, microfluidics has shed light on droplet generating/sorting where each of the formed immiscible droplets acts as an isolated reactor. The volume of droplets is within the range of nanolitre to picolitre (15 and 200 μm diameter) [2], meaning that amount of reagent and samples used are very small. Thus, experimental costs, which are highly dependent on the cost of (usually) expensive solutions, would drastically decrease. After the formation of droplets, with each containing the targeted species/cells, further steps including detection, mixing, reaction kinetic study, biochemical analysis, drug delivery and biomedical diagnosis can be executed [3].

To conduct the tests successfully, precise control over the droplet size and frequency is needed [4]. Some approaches, for instance, drug synthesis, require highly uniform droplets [5], while in some others, such as polymerase chain reaction (PCR), a variation in droplet size is more favourable [6]. Many studies have explored controlling the droplet size both experimentally and analytically in different channels [7,8]. It is known that droplet size depends on the channel’s geometrical dimensions, interfacial tension, flow rates and viscosity of the two immiscible phases [9,10,11,12]. Influenced by the mentioned parameters, four common droplet generation regimes including squeezing, dripping, jetting and tip-streaming are achieved—shown in Figure 1b. Another important criterion is wall contact angle, and the material used to fabricate the channel should be chosen accordingly. In droplet microfluidics, it is crucial for the continuous phase to wet the channel walls, which is quantified by the contact angle. If the droplets are aqueous, the surface must be hydrophobic ($\theta <90^{\circ }$) for droplets to be generated and as the hydrophobicity increases, the droplet becomes smaller. Many pieces of research are devoted to surface coating so that a desirable droplet generation is obtained [3]. Another approach toward changing the wall wettability is to add surfactants to the dispersed or continuous phase [13].

Generally, four main geometries, namely T-junction, cross-junction, flow-focusing and step-emulsification, are employed for droplet generation. Due to the simplicity of T-junction and cross-junction in geometry and, consequently, fabrication, these two have become more popular and will be discussed in the following. Initially, in 2001, Thorsen et al. [14] realised that droplets could be generated in a T-junction channel as a result of shear stress imposed by continuous phase to the dispersed one. Then, with the help of surface tension, a thread of dispersed phase was formed and eventually broken into droplets. Afterwards, in 2003, Anna et al. [15] introduced another class of geometry named flow-focusing devices. These devices offer improved monodispersity and high throughput in comparison to cross-flow geometry [5]. Cross-junction, in which the continuous phase enters the main channel through two perpendicular side channels, is a subcategory of flow-focusing devices.

To investigate the effective parameters on droplet formation, two fundamental works were conducted in 2006 by Garstecki et al. [9] and van der Graff et al. [10] who investigated droplet formation in terms of flow rate ratios and viscosity ratios. Henceforth, droplet microfluidics has further been being studied in a variety of aspects. For instance, it is reported that in flow-focusing devices the droplet size has a linear dependency on the flow rate ratio while it exhibits a power-law dependency on the capillary number ($Ca=\frac{\mu_{c}\cdot Q_{c}}{A\cdot \sigma }=\frac{\mu_{c}\times 2\times U_{c}}{\sigma }$) [11,16]. Sartipzadeh et al. [17] studied the role of cross-junction channel geometry and its aspect ratios on droplet volume and shape.

In droplet microfluidics, most of the studies have focused on Newtonian fluids, both experimentally and analytically [9,17,18]. On the contrary, a wide variety of fluids used in real life assays are of non-Newtonian behaviour, including protein, polymers and emulsions—having large and complicated molecules. Hence, for microfluidic devices to be used in such assays, it is crucial to consider non-Newtonian flows on the laboratory scale so that the outcome can be harnessed in actual practice. Among the very limited studies considering non-Newtonian fluids, almost all of them take continuous phase to be non-Newtonian.

Sang et al. [19] numerically examined the effect of Bingham and power-law fluids as the continuous phase and demonstrated that by increasing the power-law index, *n*, and the consistency coefficient, *K*, in power-law fluids, the droplet diameter would decrease while the yield stress played the dominant role in droplet size for the Bingham fluids. Sontti et al. [20,21] investigated the formation of Newtonian droplets in non-Newtonian liquid in T-junction [20] as well as cross-junction [21], and reported that rheological parameters drastically affected the droplet formation in terms of size, frequency and regime. To be more specific, they concluded that increasing the effective viscosity would raise the formation frequency while decreasing the droplet size. Chen et al. [11] studied the effect of the power-law parameters, *n* and *K*, on droplet formation and realised that *n* had a greater effect than *K* had on the droplet formation. Rostami et al. [22] reported that at low Ca and flow rate ratios, only squeezing can be achieved by Newtonian fluids while dripping can also be seen when non-Newtonian carrier fluid was used. They also elaborated that polydispersity would increase if non-Newtonian fluid is used to achieve dripping compared to Newtonian cases. Besanjideh et al. [12] investigated the addition of nanoparticles to the continuous phase, making it non-Newtonian, and reported that the droplet generation regime can be altered by changing the continuous phase from pure water to nanofluid aqueous solutions in the same velocities.

Yet, a distinct gap in studying the non-Newtonian dispersed phase presents in the literature. In the cell, molecule and protein studies, practically dispersed phase fluid—which is to be reformed into isolated microreactors—expresses non-Newtonian behaviour, and the choice of fluid type is strict so that nutrients/enzymes are available in the reaction environment [11]. For instance, to conduct the cell analysis using droplet microfluidic technology, bacteria is to be encapsulated in agarose solution, which shows a shear-thinning (Pseudoplastic) behaviour [23]. On the other hand, the continuous phase is essentially a carrier without contact with the reaction environment; thus, the limitations on choosing its type are not as restrictive and one can choose between a variety of options.

Among the very first researchers considering non-Newtonian droplets, Arratia et al. [24] experimentally studied the effect of polymeric solution molecular weight on thread’s thinning behaviour and showed that droplet formation was slower than cases with Newtonian fluids. Rostami et al. [25] experimentally showed that using non-Newtonian fluids for a dispersed phase affect the system behaviour and identified flow rates for squeezing, dripping and jetting regimes for such a system. Wong et al. [26,27] experimentally and numerically investigated a Carreau–Yasuda polymeric dispersed phase in a T-junction using the level set method in a range of polymer concentrations and concluded that droplet pinch-off time and the frequency are highly dependent on fluids’ physical properties [26,27]. Research in this field is still lacking and more analysis for different geometries and fluids using different numerical approaches are needed.

Even though there are a large number of studies on droplet-based microfluidics, this technology is not vastly used in the industry. One reason is that there are some uncertainties and inconsistencies, particularly when it comes to non-Newtonian fluids. Inspired by the lack of adequate research, the present study numerically investigates the non-Newtonian power-law droplet formation in a flow-focusing channel model while the continuous phase is Newtonian.

To do so, experimental research can be arduous and time-consuming. Moreover, being able to fully control all the environmental parameters as well as keeping the devices calibrated to have high accuracy are not easy to accomplish. Hence, a validated computational model could be harnessed to investigate the influence of different parameters on droplet formation. This way, a prior comprehension of several parameters, such as desired formation regime, droplet shape and size, would be built before actual practice. The limited available literature on the numerical study of droplet formation in flow-focusing devices utilised different interface tracking approaches, including the phase-field approach, volume-of-fluid (VOF), level set (LS) and Lattice Boltzmann Method (LBM). The VOF method, which guarantees mass conservation, is the most widely used. It also allows parallel calculation and enables studying more complex geometries. Putting all into account, the VOF method is utilised to track the development and movement of the interfaces.

In this paper, the droplet size, formation regimes and detachment time are investigated in a 2-dimensional cross-junction to measure the influence of the non-Newtonian behaviour of dispersed phase on droplet generation using VOF.

## 2. Methods

### 2.1. Governing Equations

Numerous research has benefited from the VOF method to simulate multiphase flows. It has been proved to be suitable for incompressible two-phase flows as it guarantees the conservation of mass [28,29,30]. The governing equations, namely continuity and momentum, should be solved simultaneously for the whole flow field:

(1) ∇·u=0

(2) $$\rho \left(\frac{\partial u}{\partial t}+u\cdot \nabla \;u\right)=-\nabla p+\rho g+\nabla \cdot \left[\mu \left(\nabla u+\nabla^{T}u\right)\right]+F_{\sigma }$$

where u is the velocity vector, *p* is the pressure, ρ is the density, g is the gravitational acceleration, μ is the dynamic viscosity and $F_{\sigma }$ is the Laplace pressure term accounting for surface tension forces.

The properties appearing in the momentum equation are determined by the existence of two phases (in this case dispersed and continuous ones) inside the discretised control volumes. For instance, for two phases indicated by the subscripts 1 and 2, we get

(3) $$\rho =\rho_{1}\alpha +\rho_{2}\left(1-\alpha \right)$$

in which α, volume fraction, is advected by the fluid, described as an indicator for interface capturing and can be calculated by:

(4) $$\frac{\partial \alpha }{\partial t}+\nabla \cdot \left(u\alpha \right)=0$$

and α varies between 0 and 1. If it is equal to 1, it means the fluid is a continuous phase only and vice versa. If it is not equal to 0 or 1, it indicates the interfacial region. Moreover, to gain a sharp interface and regulate the numerical diffusion, a counter-gradient transport model implemented in the α equation is modified as follows:

(5) $$\frac{\partial \alpha }{\partial t}+\nabla \cdot \left(\alpha u\right)+\nabla \cdot \left(u_{r}\left(\alpha \left(1-\alpha \right)\right)\right)=0$$

As can be seen, an artificial interface compression term is added to Equation (5)—compared to Equation (4). This term would only be active in the interface region (alpha between 0 and 1). $u_{r}$ is interface-compression velocity:

(6) $$u_{r}=u_{d}\;-\;u_{c}=min\left(C_{\alpha }\left|u\right|,\;max\left|u\right|\right)\frac{\nabla \alpha }{\left|\nabla \alpha \right|}$$

where $C_{\alpha }$ is the compression factor responsible for controlling the amount of interface compression, and it can be set between 0 and 4. Researchers have a common agreement that its value should be equal to 1 for microfluidics. Higher amounts have a negative side of empowering the non-physical spurious currents, a well-known source of error in numerical modelling of low-Ca flows [11].

Another important term in the momentum equation is $F_{\sigma }$, surface tension force, which is a volumetric force and is modelled by the continuum surface force (CSF) approach. It is calculated as:

(7) $$F_{\sigma }=\sigma \kappa \left(\nabla \alpha \right)$$

where κ is the interfacial curvature defined as:

(8) $$\kappa =\nabla \cdot \left(\frac{\nabla \alpha }{\left|\nabla \alpha \right|}\right)$$

The cause of the creation of non-physical spurious currents is the abrupt change in α over the thin interfacial region. That sudden change generates errors in calculating the normal vectors and the curvature of the interface, used to evaluate the interfacial forces. It is suggested that spurious currents can be handled by computing the interface curvature (κ) from a smoother function rather than α:

(9) $${\tilde{\alpha }}_{p}=\frac{\sum_{f=1}^{n}\alpha_{f}S_{f}}{\sum_{f=1}^{n}S_{f}}$$

in which the subscripts *p* and *f* denote the cell and face indices, respectively. The linear interpolation is used to calculate the interpolated value at the face centre. It should be noted that the smoothed alpha would be only used in calculating the curvature of the interface and for the other equations alpha itself would be used [31].

(10) $$\kappa =\nabla \cdot \left(\frac{\nabla \tilde{\alpha }}{\left|\nabla \tilde{\alpha }\right|}\right)$$

In case of non-Newtonian power-law fluids, viscosity depends on the rate of strain tensor, $\dot{\gamma }$. Two other parameters used in the power-law model are consistency coefficient (*K*) and power-law index (*n*) [11].

(11) $$\begin{array}{c} \mu \left(\dot{\gamma }\right)=K{\dot{\gamma }}^{n-1} \end{array}$$

In this work, the dispersed phase is considered as a non-Newtonian power-law fluid and its viscosity is calculated by Equation (11).

### 2.2. Numerical Setup

The numerical implementation and simulations are conducted in the open-source finite-volume libraries of *OpenFOAM* version 8. In order to simulate the droplet generation in a cross-flow channel, the *interFoam* solver, which is based on the VOF method and works by tracking the fluid–fluid interface, is employed. It applies the continuum surface force (CSF) model to capture surface tension effects. VOF was smoothed using a smoother function (see Equation (9) to reduce the parasitic currents which are non-physical velocities created as a result of numerical errors. The Pressure Implicit with Splitting of Operators (PISO) algorithm is used to couple the pressure–velocity in the momentum equation. In *fvScheme* dictionary, the temporal terms are discretised using a first-order implicit Euler scheme. Spatial discretisation is performed using second-order upwind and van Leer limiter to keep the phase fraction bounded [31]—by having the Multidimensional Universal Limiter with Explicit Solution (MULES) algorithm in *OpenFOAM*. Interpolation Schemes is set to be linear. In *controlDict*, the time step is modified by setting a fixed Courant number (Co) equal to 0.3 for the whole domain and equal to 0.15 for the interfacial area. This is specifically important because choosing higher Co causing huge errors in cases with low Ca, also being reported by other authors, all emphasising in choosing Ca≤0.3 [21,32,33].

As for boundary conditions, constant velocity is selected for all three inlets. The outlet boundary is set to be atmospheric pressure. Moreover, at the solid wall, a no-slip condition is applied while the static fluid-wall contact angle is set to be 160${}^{\circ }$. In addition, it is set for the continuous phase to completely wet the walls without the dispersed phase being spread on the walls.

It should be noted that the channel is considered two-dimensional in the simulations. This assumption is valid in microfluidics because the length of the channel is usually much longer than its width causing the flow to be negligible in the corner regions. This assumption has been validated [4,34] and used by many authors [26,35].

### 2.3. Channel Geometry

**Figure 2:**
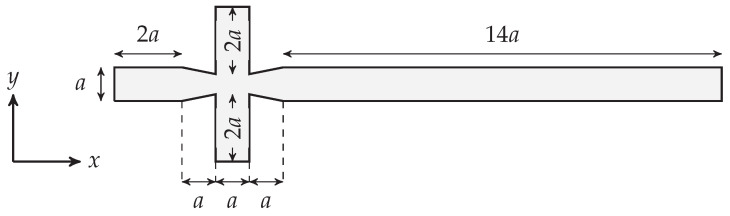
Schematic illustration of the two-dimensional channel used in this study (a=50μm).

The two-dimensional cross-junction channel used in this paper is illustrated in Figure 2. The channel has two continuous phase inlets, one dispersed phase inlet and one outlet. The lengths of three inlets are 2a—where a=50μm—each to ensure that flow is fully developed before reaching the centre of the channel. The length of the main channel is set to be 14a to capture the break-up at different locations. The slope of the contraction/expansion lines in the throat is $\frac{1}{5}$.

### 2.4. Droplet Size Calculation

**Figure 3:**
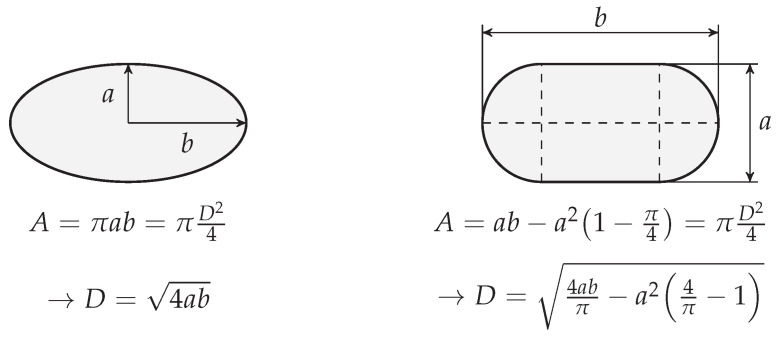
Schematic of calculating equivalent diameter, *D*, for non-spherical droplets.

In some of the simulated cases, the generated droplets are not completely spherical—see Figure 3. Hence, the surface area of that droplet is calculated by importing the results to MATLAB and then the diameter of the equivalent circle having the same surface area is reported.

### 2.5. Fluid Properties

To study the effect of non-Newtonian dispersed phase fluids on droplet generation, aqueous xanthan gum solution with different concentrations is chosen as the dispersed phase, while Newtonian oil properties are used for the continuous phase. It has been proved by other authors that power-law can model the viscosity of aqueous xanthan solutions with high accuracy [36,37]. It is worth mentioning that although the power-law model may not be applicable at low-shear rates [38,39], in confined flows the shear rate is relatively large due to the no-slip boundary condition of the walls and small dimensions. Thus, the power-law can accurately model fluid behaviour. The properties of each fluid used are listed in Table 1. The interfacial tension between the two phases is set to 0.072 N/m for all cases.

## 3. Results and Discussion

### 3.1. Mesh Convergence

**Figure 4:**
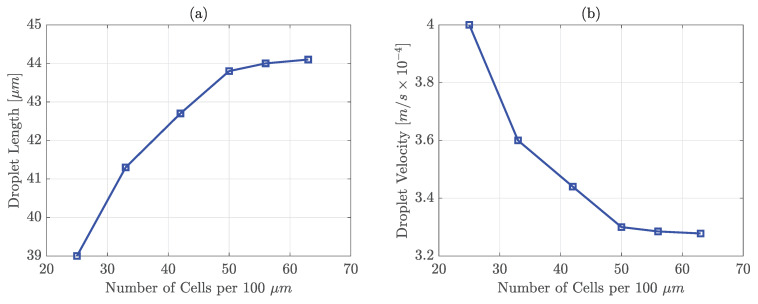
Effect of number of grids on (**a**) droplet length, (**b**) droplet velocity. The properties of continuous phase, oil, are $\mu_{c}=2.23\times 10^{-2}\;\mathrm{Pa}\cdot s$, $\rho_{c}=0.93\times 10^{3}\;{\mathrm{kg}/m}^{3}$ $U_{c}=0.064\;m/s$, and for dispersed phase, aqueous polymeric solution, viscosity calculated by power-law $\left(K=0.3125\;\mathrm{Pa}\cdot s^{n},\;n=0.389\right)$; $\rho_{d}=0.997\times 10^{3}\;{\mathrm{kg}/m}^{3}$, $U_{d}=0.0016\;m/s$; σ=0.072N/m; $\theta =160^{\circ }$.

To study the grid independence, the non-Newtonian droplet formation in the modified cross-junction is considered. The cross-junction channel domain is meshed using *blockMesh* to decompose the domain into structured hexahedral grids. To investigate the convergence, five mesh densities of $N_{x}\;\left(=N_{y}\right)=25,\;33,\;42,\;50,\;56,\;63$ cells per 100 μm or in other words, cell sizes equal to Δx(=Δy)=4,3,2,1.8,1.6 μm, respectively, are simulated and the results are compared. As it can be seen in Figure 4, large mesh sizes cause a higher droplet velocity magnitude and a lower droplet size compared to smaller mesh sizes. For $N_{x}\;\left(=N_{y}\right)\geq 50$ cells per 100 μm, droplet diameter and velocity become almost constant. Aside from that, large mesh sizes generally result in having a diffuse interface rather than a sharp one. Putting all those into account, 50 cells per 100 μm are selected for all simulations to balance accuracy and computational time.

### 3.2. Validation

**Figure 5:**
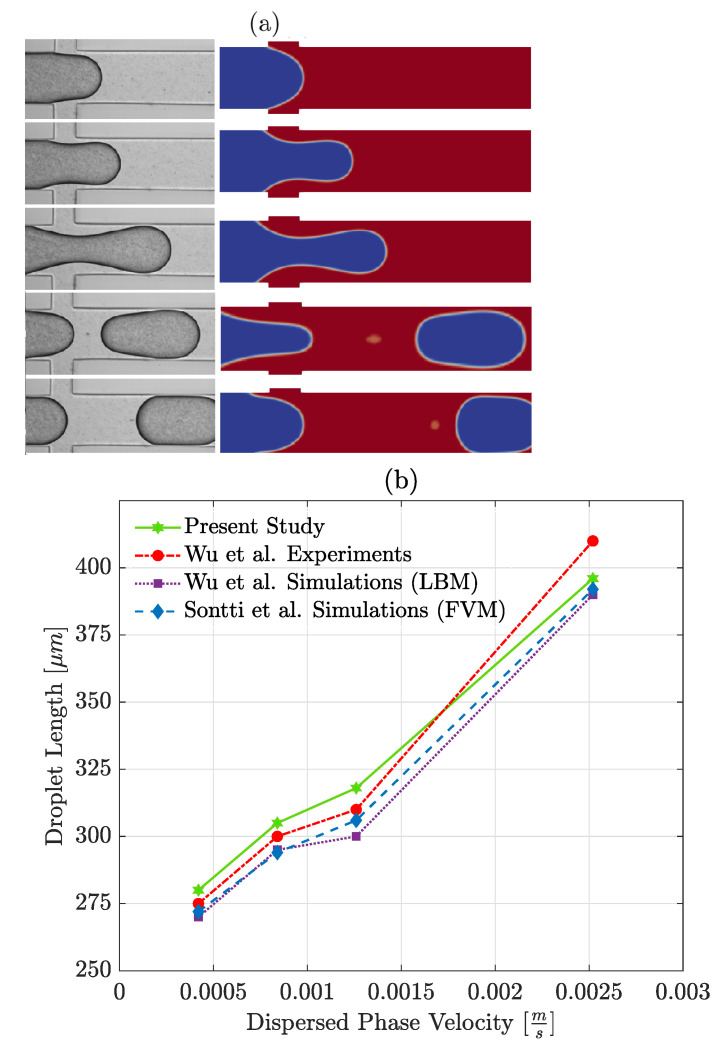
(**a**) Comparison of droplet formation steps for $\mu_{d}=1.074\times 10^{-2}\;\mathrm{Pa}\cdot s$ and $\rho_{d}=1.03\times 10^{3}\;{\mathrm{kg}/m}^{3}$, $\mu_{c}=2.441\times 10^{-2}\;\mathrm{Pa}\cdot s$ and $\rho_{c}=0.93\times 10^{3}\;{\mathrm{kg}/m}^{3}$, $U_{c}=0.00256\;m/s$, $U_{d}=0.00084\;m/s$, σ=0.03N/m; *left:* Wu et al. [41] experiments (reprinted with permission from the publisher, Elsevier), and *right:* present simulation. (**b**) Comparison of droplet length predictions against the experimental and numerical results of Wu et al. [41] and Sontti et al. [21] for $U_{c}=0.00252\;m/s$, σ=0.03N/m and $\mu_{d}/\mu_{c}=0.44$.

To ensure the simulated results are reliable, the developed model is validated against two papers. Wu et al. [41] studied water droplet generation in oil using a conventional cross-junction, both experimentally and numerically—using the Lattice Boltzmann Method (LBM). Droplet generation steps in the present study are compared with Wu et al. [41] in Figure 5a. Moreover, droplet size is compared for a constant $U_{c}=0.00252\;m/s$ and four different $U_{d}$—see Figure 5b. For low Ca cases (or low velocities), results are in excellent agreement with other works. As Ca increases, the error rises, resulting in 8% error for $U_{d}=0.00252\;m/s$. This has also been reported by other authors using VOF, stating that the error became larger (between 10 and 18%) and the droplet size was underestimated at high continuous phase flow rates [20,42]. Considering that the error in all cases is less than 8%, it is concluded that the *OpenFOAM* numerical setup is acceptable and can be used for further investigation. In the following, the effects of polymer concentration on the system’s behaviour in terms of droplet size and detachment time for a range of Ca are discussed.

As can be seen in Figure 5b, the highest deviation from the experiment was seen at the highest dispersed phase velocity or in other words, a flow rate ratio equal to 1 in different simulation methods. This may indicate that at high flow rate ratios a more refined mesh would be needed to have better results. The simulations in this work were done at a low flow rate ratio (=0.025) where the accuracy of the simulations is reliable. The droplets achieved in this study are larger than the one reported by Wu et al. [41] and Sontti et al. [20] which may be due to running the simulations in 2D and not 3D (which was the case for other studies).

### 3.3. Droplet Size

**Figure 6:**
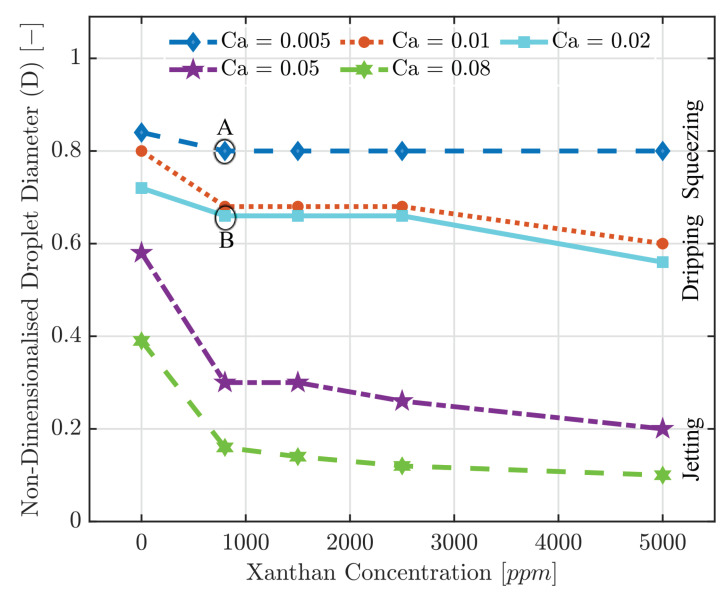
Non-dimensionalised droplet diameter versus xanthan concentration for different Ca. $C_{x}=0$ represents the droplet diameter for Newtonian deionised water droplets. Letters A and B indicate tests examined in more detail in Figure 7.

In all cases, by setting the dispersed phase viscosity proportional to shear rate, or in other dictating non-Newtonian performance, a certain decrease in droplet size for all xanthan concentrations $\left(C_{x}\right)$ was recorded—see Figure 6. This can be explained by the fact that by choosing a power-law shear thinning model for the xanthan solution (as the dispersed phase), the effective viscosity becomes larger compared to Newtonian fluid (deionised water), leading to a larger cross-flow drag force. Hence, droplet size would decrease. The amount of reduction depends on the formation regimes and $C_{x}$, and this is further explained in the following sections.

#### 3.3.1. Squeezing

When Ca is low, viscous stress is not large enough to overcome the capillary pressure. The interface between two immiscible fluids expands until it blocks the junction region, also leading to the pressure increase in the continuous phase. After the pressure reaches the maximum, the interface tension can no longer withstand the pressure; and thereby, it shrinks until droplets pinch-off. Simultaneously, the continuous phase pressure decreases. As viscous stress is not the dominant force here, viscosity values do not play a key role in droplet size, while the velocities become important. Thus, in low Ca (=0.005), the droplet size highly depends on velocities and channel geometry. Consequently, increasing the xanthan concentration did not change the droplet size as seen in Figure 6.

#### 3.3.2. Dripping

**Figure 7:**
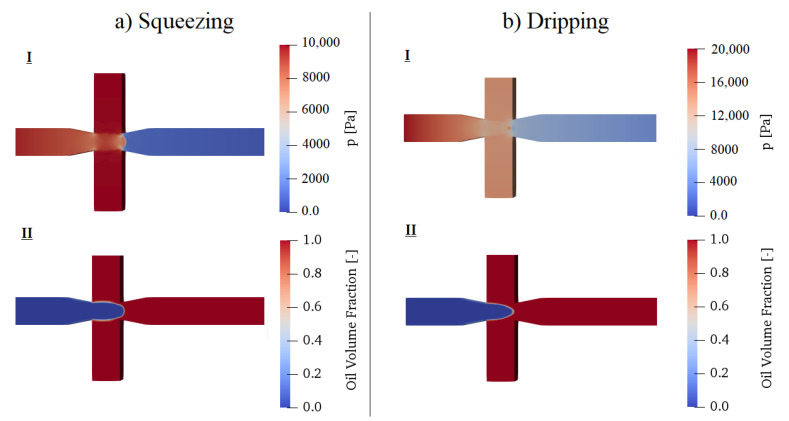
Comparison between droplet formation in (**a**) squeezing (Ca=0.005—represented by point A in Figure 6): **I.** pressure build-up at continuous phase due to junction obstruction, **II.** oil interface between the two fluids; (**b**) dripping (Ca=0.02—represented by point B in Figure 6): **I.** pressure gradient which is smoother than squeezing, **II.** oil interface between the two fluids. In both cases, $C_{x}=800\;\mathrm{ppm}$.

As the Ca increases, the droplet formation regime changes from squeezing to dripping in which the viscous effect becomes large enough to dominate over interfacial forces. As a result, the pressure difference between the two fluids develops to be more comparable. In the dripping regime, a thread of dispersed phase form within the continuous phase in the centre of the channel because shear forces are strong enough to push the interface away before giving it time to obstruct the channel. Thus, smaller droplets than the main channel entrance size can be achieved. Figure 7 gives a qualitative comparison between the pressure profiles and the amount of channel obstruction by the dispersed phase in the squeezing and dripping cases. In dripping, because the main channel entrance is not fully obstructed, a continuous phase finds its way to the main channel and a smoother pressure profile is obtained.

By setting the Ca=0.01, it was realised that in the cases of low $C_{x}$, droplet size was constant (D=0.68). However, for $C_{x}=5000\;\mathrm{ppm}$, viscosity started to affect the droplet size as shown in Figure 6 from the respective Ca. In this case, *D* was reduced to 0.6 which was 11% lower compared to other concentrations. In higher Ca (=0.02) and $C_{x}=5000\;\mathrm{ppm}$, a larger reduction of *D*, equal to 15%, is seen, which is the result of stronger impacts of viscous shear force.

#### 3.3.3. Jetting

Generally, by an increase in fluids flow rate (both continuous and dispersed phases), the dripping regime is switched to jetting. In this study, both velocities were increased to switch from one formation regime to another while keeping the flow rate ratio constant. In the jetting regime, the deformation of threads is a result of Rayleigh–Plateau instabilities. Generally, much smaller droplets than channel width (as well as the ones formed in squeezing and dripping) with diameters equal to a few microns are formed. In this regime, the effect of viscosity becomes more apparent and droplet size is influenced by it even in medium $C_{x}$. When Ca=0.005, *D* for $C_{x}=2500$ and 5000ppm has decreased to 0.26 and 0.2, respectively. Further increase in the Ca results in having such effect even in lower $C_{x}$ starting from 800ppm—as depicted in Figure 6. For Ca=0.08, if the dispersed phase is considered to be Newtonian, droplets with two diameters equal to 25μm and 14μm were generated simultaneously. Such polydispersity is seen and reported by other authors as well [13]. It was observed that by choosing a non-Newtonian fluid, such polydispersity vastly reduced and droplets’ size could be considered almost constant.

**Figure 8:**
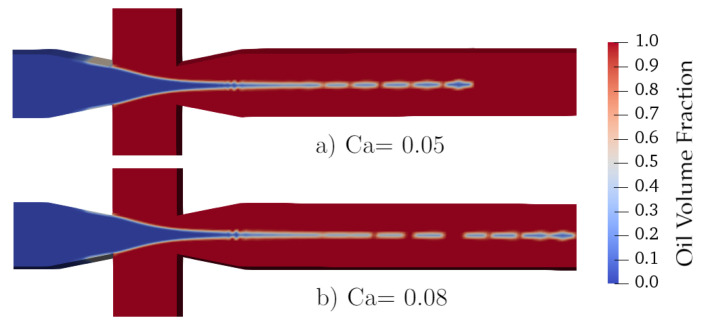
Transition from jetting toward tip streaming at high xanthan concentration for two Ca; (**a**) Ca=0.05, (**b**) Ca=0.08.

One observation specific to this section is that for both Ca=0.05 and Ca=0.08 with the highest $C_{x}$, the droplet formation regime becomes more like tip streaming. For those cases as very small droplets (10μm and 4μm) are detached from an elongated thread as illustrated in Figure 8. A thread of dispersed phase penetrates into the main channel, and then a large number of very small droplets are generated at its tip. These droplets are not spherical and tend to be like thin plugs which are again due to very high viscosity, not letting the droplets to reform.

Note that in all the mentioned regimes above, the shear rate inside the non-Newtonian droplet was within the range of 20 to $10^{4}$ ensuring that the power-law is capable of modelling the fluid behaviour inside the channel [37].

### 3.4. Detachment Time

**Figure 9:**
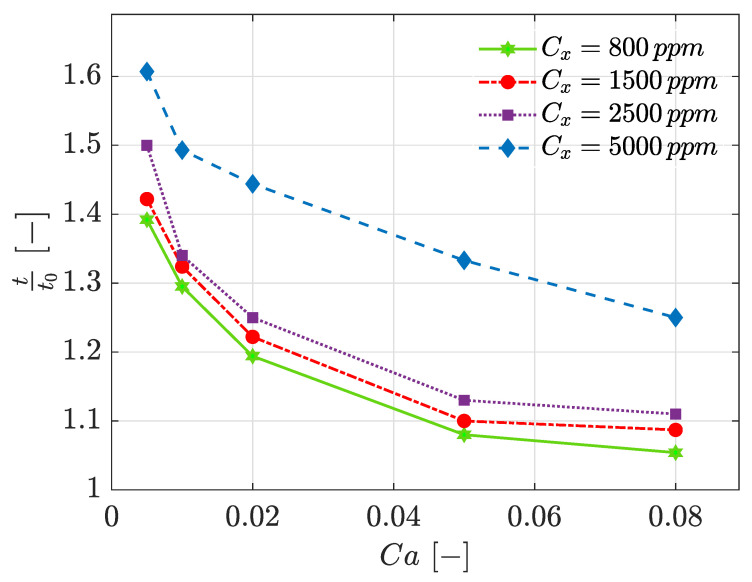
Non-dimensionalised droplet formation time versus Ca for different xanthan concentrations.

Aside from the droplet size, detachment time is another parameter affected by the non-Newtonian behaviour as well as Ca. Such effect was studied for four xanthan concentrations and four Ca and the results were shown in Figure 9. Note that the *y*-axis of Figure 9 represents the non-dimensionalised droplet formation time, which is equal to the studied case droplet formation time divided by Newtonian droplet formation time—e.g., $C_{x}=0\;\mathrm{ppm}$—for that specific Ca.

It can be understood from Figure 9 that in all xanthan concentrations, $t/t_{0}$ is higher than 1. This means that in all cases, droplet formation required more time when compared to the case with the Newtonian dispersed phase. Moreover, for constant Ca, increasing the xanthan concentration results in a larger deviation from Newtonian cases ($t/t_{0}$ increases). Higher apparent viscosity causes the thread to tend to elongate rather than break, and hence, the necking stage would take a longer time. Furthermore, the biggest delays are detected in squeezing and where Ca increases, resulting in smaller delay. At low Ca, surface tension forces are stronger, and so, the fluid would be more resistant to detachment. Consequently, droplet generation in squeezing would be more time consuming compared to other cases.

## 4. Conclusions

In this work, the generation of non-Newtonian droplets within the Newtonian continuous phase through a cross-junction microfluidic channel with a square cross-section was numerically analysed using *OpenFOAM* version 8. For that aim, the impact of non-Newtonian power-law xanthan gum solution as the dispersed phase in a range of concentrations on droplet generation was studied and compared with Newtonian fluids. Increasing the xanthan gum concentration changes the rheological behaviour of the dispersed phase by increasing the apparent viscosity and degree of shear-thinning. It was found that:

- Non-Newtonian behaviour affected the droplet size and the influence became more apparent as the flow deviated more from being Newtonian.
- The droplet size reduced in all cases for all regimes compared to Newtonian fluid due to higher apparent viscosity.
- In the squeezing regime, altering xanthan concentration did not change the droplet diameter because viscous stress is not the dominant force in this regime. Droplets form as a result of capillary pressure and channel blockage by the dispersed phase.
- In higher Ca, high concentrations of polymer ($C_{x}>2500\;\mathrm{ppm}$) caused a further reduction in droplet size, and this is due to the dominant influence of viscous forces (large apparent viscosity).
- At Ca where jetting occurs for a Newtonian fluid, changing fluid rheology to highly shear-thinning behaviour will alter the droplet regime to tip-streaming.
- As concentration increases, an elongated dispersed thread is formed and penetrates further inside the continuous phase as a result of high viscous pressure. Consequently, the droplet breakup point moves toward downstream and the droplet pinch-off is delayed.

This research highlights the potential to control the droplet size and formation regime by administering the shear-thinning behaviour of the dispersed phase through the choice of polymer concentration. Further investigation of the effect of other parameters, including stabiliser addition and flow rate ratio alternation, on shear-thinning as well as shear-thickening droplets would provide more understanding on droplet formation in real-life assays.

## Figures and Tables

**Table 1:** Physical properties of the fluids used and power-law parameters of the xanthan aqueous solutions.

Fluid	μ [Pa·s]	ρ [kg/m^3^]	*n*	*K* [Pa·s^n^]	ref.
Water	0.00089	997	—	—	—
Oil	0.0223	930	—	—	—
800 ppm xanthan/water	—	997	0.491	0.0755	[40]
1500 ppm xanthan/water	—	997	0.389	0.3125	[40]
2500 ppm xanthan/water	—	997	0.302	0.985	[40]
5000 ppm xanthan/water	—	997	0.16	10.538	[25]

## Data Availability

The data presented in this study are available on request from the corresponding author.

## References

[1] Whitesides G.M. (2006). The origins and the future of microfluidics. Nature.

[2] Leman M., Abouakil F., Griffiths A.D., Tabeling P. (2015). Droplet-based microfluidics at the femtolitre scale. Lab Chip.

[3] Samiei E., Tabrizian M., Hoorfar M. (2016). A review of digital microfluidics as portable platforms for lab-on a-chip applications. Lab Chip.

[4] Anna S.L. (2016). Droplets and bubbles in microfluidic devices. Annu. Rev. Fluid Mech..

[5] Sesen M., Alan T., Neild A. (2017). Droplet control technologies for microfluidic high throughput screening (*μ*HTS). Lab Chip.

[6] Zeng Y., Shin M., Wang T. (2013). Programmable active droplet generation enabled by integrated pneumatic micropumps. Lab Chip.

[7] Brenker J.C., Collins D.J., Van Phan H., Alan T., Neild A. (2016). On-chip droplet production regimes using surface acoustic waves. Lab Chip.

[8] Sánchez Barea J., Lee J., Kang D.K. (2019). Recent advances in droplet-based microfluidic technologies for biochemistry and molecular biology. Micromachines.

[9] Garstecki P., Fuerstman M.J., Stone H.A., Whitesides G.M. (2006). Formation of droplets and bubbles in a microfluidic T-junction—scaling and mechanism of break-up. Lab Chip.

[10] Van der Graaf S., Nisisako T., Schroën C., Van Der Sman R., Boom R. (2006). Lattice Boltzmann simulations of droplet formation in a T-shaped microchannel. Langmuir.

[11] Chen Q., Li J., Song Y., Christopher D.M., Li X. (2020). Modeling of Newtonian droplet formation in power-law non-Newtonian fluids in a flow-focusing device. Heat Mass Transf..

[12] Besanjideh M., Shamloo A., Kazemzadeh Hannani S. (2021). Enhanced oil-in-water droplet generation in a T-junction microchannel using water-based nanofluids with shear-thinning behavior: A numerical study. Phys. Fluids.

[13] Zhu P., Wang L. (2017). Passive and active droplet generation with microfluidics: A review. Lab Chip.

[14] Thorsen T., Roberts R.W., Arnold F.H., Quake S.R. (2001). Dynamic pattern formation in a vesicle-generating microfluidic device. Phys. Rev. Lett..

[15] Anna S.L., Bontoux N., Stone H.A. (2003). Formation of dispersions using “flow focusing” in microchannels. Appl. Phys. Lett..

[16] Fu T., Wu Y., Ma Y., Li H.Z. (2012). Droplet formation and breakup dynamics in microfluidic flow-focusing devices: From dripping to jetting. Chem. Eng. Sci..

[17] Sartipzadeh O., Naghib S.M., Seyfoori A., Rahmanian M., Fateminia F.S. (2020). Controllable size and form of droplets in microfluidic-assisted devices: Effects of channel geometry and fluid velocity on droplet size. Mater. Sci. Eng. C.

[18] Soh G.Y., Yeoh G.H., Timchenko V. (2016). Improved volume-of-fluid (VOF) model for predictions of velocity fields and droplet lengths in microchannels. Flow Meas. Instrum..

[19] Sang L., Hong Y., Wang F. (2009). Investigation of viscosity effect on droplet formation in T-shaped microchannels by numerical and analytical methods. Microfluid. Nanofluid..

[20] Sontti S.G., Atta A. (2017). CFD analysis of microfluidic droplet formation in non–Newtonian liquid. Chem. Eng. J..

[21] Sontti S.G., Atta A. (2019). Numerical insights on controlled droplet formation in a microfluidic flow-focusing device. Ind. Eng. Chem. Res..

[22] Rostami B., Morini G.L. (2020). Generation of Newtonian droplets in Newtonian and non-Newtonian carrier flows in micro T-junctions under opposed-flow configuration. J. Non-Newton. Fluid Mech..

[23] Hussein M.H. (2015). Extraction of Agar from Gelidium P (Rhodophyta) and Green Synthesis of Agar/Silver Nanoparticles. J. Agric. Chem. Biotechnol..

[24] Arratia P.E., Cramer L., Gollub J.P., Durian D.J. (2009). The effects of polymer molecular weight on filament thinning and drop breakup in microchannels. New J. Phys..

[25] Rostami B., Morini G. (2017). Micro droplets of non-Newtonian solutions in silicone oil flow through a hydrophobic micro cross-junction. J. Phys. Conf. Ser..

[26] Wong V.L., Loizou K., Lau P.L., Graham R.S., Hewakandamby B.N. (2017). Numerical studies of shear-thinning droplet formation in a microfluidic T-junction using two-phase level-SET method. Chem. Eng. Sci..

[27] Wong V.L., Loizou K., Lau P.L., Graham R.S., Hewakandamby B.N. (2019). Characterizing droplet breakup rates of shear-thinning dispersed phase in microreactors. Chem. Eng. Res. Des..

[28] Wang M., Kong C., Liang Q., Zhao J., Wen M., Xu Z., Ruan X. (2018). Numerical simulations of wall contact angle effects on droplet size during step emulsification. RSC Adv..

[29] Wu L., Liu X., Zhao Y., Chen Y. (2017). Role of local geometry on droplet formation in axisymmetric microfluidics. Chem. Eng. Sci..

[30] Kobayashi I., Mukataka S., Nakajima M. (2004). CFD simulation and analysis of emulsion droplet formation from straight-through microchannels. Langmuir.

[31] Hoang D.A., van Steijn V., Portela L.M., Kreutzer M.T., Kleijn C.R. (2013). Benchmark numerical simulations of segmented two-phase flows in microchannels using the Volume of Fluid method. Comput. Fluids.

[32] Biroun M., Rahmati M., Jangi M., Tao R., Chen B., Fu Y. (2019). Computational and experimental analysis of droplet transportation/jetting behaviours driven by thin film surface acoustic waves. Sens. Actuators A Phys..

[33] Mora A.E.M., de Lima e Silva A.L.F., de Lima e Silva S.M.M. (2019). Numerical study of the dynamics of a droplet in a T-junction microchannel using OpenFOAM. Chem. Eng. Sci..

[34] Hoang D., Portela L., Kleijn C., Kreutzer M., Van Steijn V. (2013). Dynamics of droplet breakup in a T-junction. J. Fluid Mech..

[35] Liu H., Zhang Y. (2009). Droplet formation in a T-shaped microfluidic junction. J. Appl. Phys..

[36] Song K.W., Kim Y.S., Chang G.S. (2006). Rheology of concentrated xanthan gum solutions: Steady shear flow behavior. Fibers Polym..

[37] Wyatt N.B., Liberatore M.W. (2009). Rheology and viscosity scaling of the polyelectrolyte xanthan gum. J. Appl. Polym. Sci..

[38] Picchi D., Ullmann A., Brauner N. (2018). Modeling of core-annular and plug flows of Newtonian/non-Newtonian shear-thinning fluids in pipes and capillary tubes. Int. J. Multiph. Flow.

[39] Picchi D., Ullmann A., Brauner N., Poesio P. (2021). Motion of a confined bubble in a shear-thinning liquid. J. Fluid Mech..

[40] Taassob A., Manshadi M.K.D., Bordbar A., Kamali R. (2017). Monodisperse non-Newtonian micro-droplet generation in a co-flow device. J. Braz. Soc. Mech. Sci. Eng..

[41] Wu L., Tsutahara M., Kim L.S., Ha M. (2008). Three-dimensional lattice Boltzmann simulations of droplet formation in a cross-junction microchannel. Int. J. Multiph. Flow.

[42] Li X., He L., He Y., Gu H., Liu M. (2019). Numerical study of droplet formation in the ordinary and modified T-junctions. Phys. Fluids.

